# The feasibility and acceptability of conducting a trial of specialist medical care and the Lightning Process in children with chronic fatigue syndrome: feasibility randomized controlled trial (SMILE study)

**DOI:** 10.1186/1745-6215-14-415

**Published:** 2013-12-05

**Authors:** Esther Crawley, Nicola Mills, Lucy Beasant, Debbie Johnson, Simon M Collin, Zuzana Deans, Kate White, Alan Montgomery

**Affiliations:** 1 Centre for Child & Adolescent Health, School of Social and Community Medicine, University of Bristol, Oakfield House, Oakfield Road, BS8 2BN, Bristol, UK; 2 School of Social and Community Medicine, University of Bristol, Canynge Hall, Bristol BS8 2PS, UK; 3 Nottingham Clinical Trials Unit, Queen’s Medical Centre, Nottingham NG7 2UH, UK

## Abstract

**Background:**

Chronic fatigue syndrome (CFS) or myalgic encephalomyelitis (ME) is relatively common in children with limited evidence for treatment. The Phil Parker Lightning Process (LP) is a trademarked intervention, which >250 children use annually. There are no reported studies investigating the effectiveness or possible side effects of LP.

**Methods:**

The trial population was drawn from the Bath and Bristol NHS specialist paediatric CFS or ME service. The study was designed as a pilot randomized trial with children (aged 12 to 18 years) comparing specialist medical care with specialist medical care plus the Lightning Process. Integrated qualitative methodology was used to explore the feasibility and acceptability of the recruitment, randomization and interventions.

**Results:**

A total of 56 children were recruited from 156 eligible children (1 October 2010 to 16 June 2012). Recruitment, randomization and both interventions were feasible and acceptable. Participants suggested changes to improve feasibility and acceptability and we incorporated the following in the trial protocol: stopped collecting 6-week outcomes; introduced a second reminder letter; used phone calls to collect primary outcomes from nonresponders; informed participants about different approaches of each intervention and changed our recommendation for the primary outcome for the full study from school attendance to disability (SF-36 physical function subscale) and fatigue (Chalder Fatigue Scale).

**Conclusions:**

Conducting randomized controlled trials (RCTs) to investigate an alternative treatment such as LP is feasible and acceptable for children with CFS or ME. Feasibility studies that incorporate qualitative methodology enable changes to be made to trial protocols to improve acceptability to participants. This is likely to improve recruitment rate and trial retention.

**Trial registration:**

**Feasibility study first randomization:** 29 September 2010.

**Trial registration:** Current Controlled Trials ISRCTN81456207 (31 July 2012).

**Full trial first randomization:** 19 September 2012.

## Background

Chronic fatigue syndrome or myalgic encephalomyelitis (CFS/ME) in children is relatively common, affecting between 0.1 and 2% of secondary school children [1-5]. It is potentially serious, with over 50% of children bed-bound at some stage and an average school absence of one academic year [6,7]. CFS/ME is defined as ‘generalized fatigue, causing disruption of daily life, persisting after routine tests and investigations have failed to identify an obvious underlying “cause”’ [8]. National Institute of Health & Clinical Excellence (NICE) guidelines recommend a minimum duration of 3 months of fatigue before making a diagnosis in children [9].

Specialist medical care for CFS/ME follows evidence-based approaches as recommended in NICE guidelines [9]. This evidence includes two randomized controlled trials (RCTs) comparing cognitive behavioural therapy with waiting list (delayed cognitive behavioural therapy) [10,11] or internet-based CBT with normal care [12], one trial comparing family-focused cognitive behavioural therapy with psycho-education [13] and one observational study comparing outcomes in children who chose outpatient multidisciplinary rehabilitative treatment (graded activities or exercises and supportive care) instead of supportive care alone [14].

The Phil Parker Lightning Process® (LP) is a trademarked intervention that is used for a variety of conditions including CFS/ME. It was developed from osteopathy, life coaching and neurolinguistic programming. The LP trains individuals to recognize when they are stimulating or triggering unhelpful physiological responses and to avoid these, using a set of standardized questions, new language patterns and physical movements with the aim of improving a more appropriate response to situations (http://www.lightningprocess.com). The intervention includes three group sessions on consecutive days where participants are taught theories and skills, which are then practised through simple steps, posture and coaching. Families currently pay approximately £620 to attend the Lightning Process course. Even though more than 250 children per year use the LP as an intervention for their CFS/ME, there are currently no reported studies investigating the effectiveness or possible side effects of LP in children.

Feasibility studies are used to estimate important study variables, for example standard deviation of the outcome measure, the willingness of participants to be randomized and the number of eligible patients [15]. Recruitment can be improved by audio-recording recruitment consultations, evaluating information exchange, and retraining recruiters to improve collection of informed consent, rates of randomization and acceptance of allocation [16,17]. In this study, we report on the feasibility and acceptability of recruiting families into a trial involving an alternative intervention (the Lightning Process) for CFS/ME to inform the design of a full-scale, adequately powered RCT comparing LP with LP plus specialist medical care.

## Method

### Population

Children were recruited between October 2010 and June 2012 at initial clinical assessment appointments conducted by the Bath and Bristol specialist paediatric CFS/ME service. This service provides assessment and treatment for more than 250 children each year. Most patients are from South Gloucestershire, Bristol, Somerset and Wiltshire, but children and young people from across the UK also receive assessment and treatment. Children were eligible for this study if they were diagnosed with CFS/ME according to NICE diagnostic criteria [9], they were mildly to moderately affected (able to attend clinic appointments), were 12 to 18 years old and were sufficiently literate in the English language to understand the patient information sheet.

**Figure 1:**
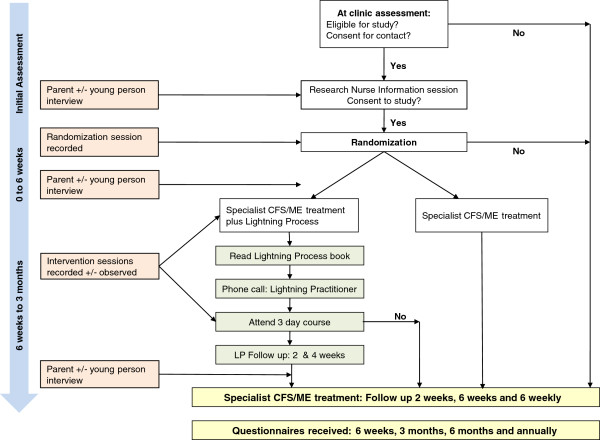
Protocol flow chart.

Figure 1 describes the recruitment process. Eligible children and their families were identified by the clinician conducting the assessment. If the child and his or her family were willing to find out more about the study a researcher contacted the family and arranged to visit them at a convenient location (usually at home) to discuss and provide further information, including: the study rationale, the uncertainties about the effectiveness of either intervention, the known advantages or disadvantages of the interventions, the options available outside the RCT, and the right not to take part or withdraw at any time. Those willing to take part were randomized to receive either specialist medical care or to specialist medical care plus the Phil Parker Lightning Process (LP). Allocation was minimized [18] by sex and age retaining a probabilistic element using a computer-generated random number sequence, and was implemented using an automated telephone randomization service provided by the Bristol Randomized Trials Collaboration to ensure concealment from clinical staff undertaking recruitment.

### Interventions

#### Specialist medical care

Children and their families were offered a variety of treatment options [9] centred around graded activity. Participants typically received a follow-up phone call 2 weeks after assessment followed by family-based rehabilitation consultations at approximately 6 weeks, 3 months and 4.5 months (each lasting one hour). The number and timing of the sessions were agreed with the child and family, and varied depending on the needs and goals of the child. Other interventions, such as cognitive behavioural therapy or graded exercise therapy (GET), were offered to children if needed (usually if there were comorbid mood problems or the primary goal was sport-related, respectively).

#### Specialist medical care plus the Lightning Process

In addition to specialist medical care, children and their parents in this arm were asked to read information about the Lightning Process on the internet. They then followed the usual LP procedure (reading the introductory LP book or listening to it in CD form) and completing an assessment form to identify goals and describe what was learnt from the book. On receiving completed forms, an LP practitioner telephoned the children to check whether they were ready to attend an LP course. The courses were run with two to four children over three sessions (each 3 hours 45 minutes) on three consecutive days. During the group sessions, children had a theory session (elements of the stress response, how the mind and body interact and how thought processes can be helpful or negative) and a practical session to put skills into practice. Children and their families then had two follow-up phone calls with the LP practitioner within 2 weeks of and 6 to 8 weeks after the LP course. The purpose of the follow-up was to provide support and to check that the participant had understood what was covered on the course, were able to apply the tools and could identify when they needed to use LP, and to clarify and discuss areas of uncertainty.

### Outcome assessment

The following inventories were completed by children just before their clinical assessment (baseline) and follow-up (6 weeks and 3, 6 and 12 months): 11-item Chalder Fatigue Scale [19]; visual analogue pain rating scale; the SF-36 [20]; the Spence Children’s Anxiety Scale [21]; the Hospital Anxiety and Depression Scale (HADS) [22], a single-item inventory on school attendance and the EQ-5D five-item quality-of-life questionnaire [23]. At the start of the study, follow-up times were 6 weeks and 3, 6 and 12 months. We estimate that these questionnaires take approximately 20 to 30 minutes to complete. However, the 6-week follow-up was dropped following feedback from parents about the excessive burden of completing questionnaire packs.

Parents and carers were asked to complete the following questionnaires at baseline: socioeconomic status (education and employment), an adapted 4-item Work Productivity and Activity Impairment and General Health (WPAI:GH) questionnaire [24] and an adapted existing health resource-use questionnaire, which asked parents about the health service (for example, GP or specialist care), educational service (for example, school counsellor) and travel costs most relevant to the CFS/ME population. The latter two questionnaires were also completed at 3, 6 and 12 months follow-up.

Reminders were sent out at 2 weeks if follow-up questionnaires had not been returned. To improve follow-up rates we sent out a reduced set of questionnaires (SF-36, Chalder Fatigue Scale and school attendance question) with the reminder letter two weeks after the initial questionnaires were sent. We introduced a phone call to nonresponders two weeks after the reminder was sent, to complete the reduced questionnaire set over the telephone.

### Qualitative research

In-depth interviews were undertaken with parents on three occasions (after assessment but prior to randomization, after randomization, and after the intervention) to form ‘case studies’. Children were interviewed once at one of these times. We used purposive sampling to ensure that interviews included a range of informants, in terms of socioeconomic circumstances, age, sex, ethnicity and families from both intervention arms (maximum variation sampling) [25].

Interviews were conducted at a location of the parent’s choice and were semistructured following a checklist of topics to ensure consistency, but flexible enough to allow parents and children to raise issues of importance. Interviews explored the recruitment process; the provision of study and intervention information; participants’ beliefs, expectations, preferences and experiences of the interventions; reasons for accepting or declining participation; and the acceptability of the outcome measures. Interviews lasted approximately 20 to 60 minutes with parents and 20 minutes with children. All interviews were audio-recorded with consent, transcribed verbatim, and anonymized.

Observations (followed by discussions with a researcher) were made of children and parents completing the questionnaires at follow-up to highlight any difficulties or misunderstandings [26]. These children were recruited opportunistically when the study team was aware that a questionnaire was due for completion. Participants were observed completing follow-up questionnaires in their own homes.

All recruitment consultations were audio-recorded to document the interaction between recruiter and potential participant, so as to explore information provision, recruitment techniques, patient treatment preferences and randomization decisions and to identify recruitment difficulties and support change [16,17].

### Data analysis

We recorded the number of potentially eligible participants attending the clinic, the number assessed for eligibility, and the number of eligible patients who consented and were randomized. We compared characteristics of eligible patients who were and were not randomized using appropriate descriptive statistics (that is, mean and standard deviation or median and interquartile range (given as first and third quartile, Q1, Q3) for continuous, and number and percent for categorical variables respectively). As the aim of this study was to assess the feasibility of a future definitive trial, we did not undertake a formal sample size calculation.

Qualitative data analysis was an ongoing and iterative process, commencing soon after data collection, and informed further sampling and data collection. Interview transcripts and observation notes were imported into NVivo (version 9) where they were systematically assigned codes and analyzed thematically to identify themes using techniques of constant comparison [27]. Individuals exhibiting contrasting attitudes (‘negative cases’) were studied in detail to understand reasons underlying such contrasts and to gain a deeper understanding of the data and findings [28]. Recruitment to trial consultations were purposefully selected for analysis according to whether or not the study participant accepted randomization, different times in the study, and those that highlighted issues of study acceptability (intervention crossover and study withdrawal). Recruitment to trial consultations was analyzed for content and presentation of information relating to the interventions using techniques of content analysis [29]. Two members of the research team analyzed approximately 10% of the qualitative data independently to compare coding and enhance dependability of findings. Descriptive accounts were produced, and theoretical explanations for behaviours, opinions and decisions developed.

### Serious adverse events

Serious adverse events were reported by clinicians from the clinical team or members of the research team to the principle investigator and the sponsor within 24 hours. All serious adverse events were reviewed by the research and development committee.

### Ethical review

A favourable ethical opinion was given on the 8th September 2010, reference 10/H0206/32, by South West 2 Local Research Ethics Committee. A favourable ethical opinion was given on 31 May 2011 for an amendment to study documents and protocol.

## Results

### Feasibility of recruitment

**Figure 2:**
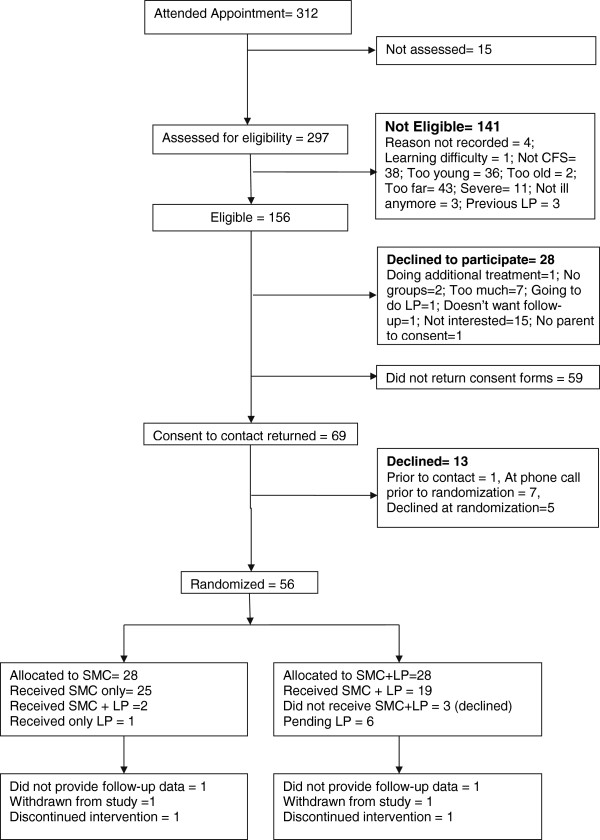
CONSORT diagram showing flow through study for patients assessed for eligibility (1 October 2010 to 16 June 2012).

Figure 2 describes the flow of children through the study. Between 1 October 1 2010 and 17 June 2012, a total of 312 children attended clinic appointments. Of the 297 assessed for eligibility, 141 were ineligible. The majority (38) did not have CFS/ME, lived too far away for follow-up (43) or were too young (36). Of the 157 eligible children, 28 declined to participate at the clinical assessment. The majority were ‘not interested’ (15) or said it was ‘too much’ (7). Fifty-nine families were given study information sheets at the clinic appointment but did not return them and it was assumed that they did not want to take part in the study.

Sixty-nine families agreed to further contact from the research officer and of these 13 children declined randomization. Seven declined further involvement prior to the recruitment to trial consultation and five declined participation at or after the consultation. The number of eligible children who were randomized was therefore 56/156 (35.9%).

Table 1 shows that the baseline characteristics of the children who were recruited and were not recruited were similar. The mean age of recruited children was 14.8 years, 76.4% were girls and the median time from onset of illness to assessment was 12 months.

**Table 1:** Characteristics of eligible patients who were and were not recruited

	**Recruited to SMILE *n*** **= 56**	**Eligible but not recruited *n*** **= 100**
	**Mean (standard deviation)**	**Mean (standard deviation)**
Age (years)	14.8 (1.6)	14.8 (1.6)
Female	42 (76.4%)	71 (73.2%)
	**Median (Q1 to Q3)**	**Median (Q1 to Q3)**
Time to assessment (months)	12 (7 to 21)	12 (6 to 24)
Chalder fatigue score (0 to 33)	25 (23 to 27)	25 (21 to 28)
SF-36 physical function (0 to 100)	55 (44 to 65)	55 (35 to 70)
Anxiety (Spence Children’s Anxiety Scale) (0 to 90)	35 (21 to 52)	33 (21 to 46)
Number of symptoms (0 to 14)	9 (7 to 10)	9 (7 to 10)
Anxiety (HADS) (0 to 21)	10.5 (7.5 to 13)	9 (7 to 12)
Depression (HADS) (0 to 21)	8 (6 to 10)	8 (5 to 11)
Visual analogue pain	52 (35 to 73)	49 (15 to 68)
School attendance in the previous week:	***n *** **(%)**	***n *** **(%)**
None	5 (9.3%)	24 (24.0%)
10%	7 (13.0%)	4 (4.0%)
20%	4 (7.4%)	6 (6.0%)
40%	9 (16.7%)	12 (12.0%)
60%	13 (24.1%)	28 (28.0%)
80%	13 (24.1%)	21 (21.0%)
100%	3 (5.6%)	3 (3.0%)
Not applicable	0 (0.0%)	2 (2.0%)

HADS, Hospital Anxiety and Depression Scale.

### Acceptance of treatment arm

All 56 participants accepted the allocation at the randomization appointment. From these, 47/56 received the treatment as allocated. During the study, 3/28 (10.7%) in the specialist medical care plus LP arm did not receive LP (one decided not to proceed after reading further information and two were unable to attend the course). Six participants were pending LP treatment at the time of analyses. We were aware that three families in the specialist medical care arm subsequently went on to obtain the LP privately and therefore did not receive this allocation. One of these families did not have specialist medical care.

### Follow-up rates

In the first 12 months of the study, we followed up 10/18 families (55.6%) at 6 months. We then implemented changes to the protocol to improve follow-up rates including a phone call to nonresponders to collect the primary and secondary outcome measures (SF-36 and Chalder Fatigue Scale). This was successful and from March 2011 to December 2011, follow-up rates of the 24 children assessed prior to January 2012 who should have had 6 month follow-up data by July 2012 were 22/24 (91.7%).

### Qualitative research findings

#### Participants

Thirteen mothers and twelve children were interviewed. One mother interviewed did not consent to her child being interviewed and two children (a sixteen-year-old boy and a fourteen-year-old girl) declined an interview. Five mothers were interviewed at all three times to form case studies. The remaining eight mothers took part in one-off interviews, four after randomization and four after the intervention. Of the twelve children interviewed, five were interviewed after randomization and seven after the intervention. Three of the children were boys and nine were girls. Seven children in the qualitative sample were randomized to the specialist medical care and Lightning Process arm and six to the specialist medical care arm of the study.

Ten further participants were observed completing questionnaires, and were asked to give verbal feedback on questionnaire acceptability as they progressed through the questionnaire. Questionnaire observations were conducted with four mothers, one father and five children (four boys and one girl).

#### Acceptability of study discussion at clinic

Most families found the initial discussion in clinic about the study acceptable. Families recognized that time was limited at the end of the clinical consultation, and some families appreciated taking the information sheets home to read because they had already received a large amount of clinical information about their child’s CFS/ME.

Parent (P)4: ‘I think we got what we needed, we just got the basics and I’m always quite happy to contribute to research if we can, if it helps’. (Prior to randomization)

Young Person (YP)88: ‘I think also we didn’t get much [oral] information. [Researcher: Did she give you the information to take?] Yes, to take with us because we were, like, running over time, so she said, ‘Have this,’ and we just went. We didn’t look at it until quite a bit after’. (Post randomization)

#### Study information sheets

Study information sheets were largely viewed by mothers as being informative and understandable. The flow chart diagram and data tables included in the information sheet were perceived as positive.

P4: ‘The diagrams really, really breaks down what the process is; it makes it very easy to see… that would be more than enough information to make a decision on, and it answers all the questions about privacy and what the purpose is and what’s going to happen’. (Post randomization)

One parent (P5) felt that although the information sheet provided, ‘everything they needed to know,’ it was at the same time slightly overwhelming:

P5: ‘I think it should be simplified, because I think it is quite scary to read the pack with the interviews; it is quite intense and it was quite off-putting, to be honest’. (Prior to randomization)

Most children relied on their parent’s verbal explanation of the patient information sheet, especially those aged 12 to 14 years, primarily because it was perceived as long, difficult to understand, repetitive in places and not visually appealing to 12 to 18 year olds.

YP38: ‘I thought they were quite long, given that we’re reading about CFS and I find it very hard to concentrate so it took me a long time to read through the whole thing because I had to read it through quite a few times to actually understand it… and I actually ended up asking Mummy to read it herself and then tell me verbally; like, sum it up to me verbally, which was better, so that I could really understand… other than the graphs at the end, I didn’t get, but Mummy then worked them out’. (Post randomization)

YP35: ‘I think that when you look at it you don’t … it doesn’t look that interesting so … it makes you not interested in reading it’. (Post randomization)

A few children felt the term ‘interviews’ was too formal and somewhat ‘scary’, reminding them of a formal interview situation where they would be put on the spot.

YP22: ‘Because of all the different interviews with all the different people: she said I had to have a few 20-minute interviews with different people and I’m not very confident, so…’. (Prior to randomization)

#### Recruitment consultations

Ten audio-recordings of recruitment consultations were analyzed. Consultations were good in terms of the provision of study information and were conducted at a good pace with a good rapport. However, discussion of the interventions tended to be weighted towards the Lightning Process rather than the specialist medical care. This was fed back to the research officer through written information and discussion and suggestions were made to redress the balance and incorporated in future recruitment consultations.

The majority of families were positive about the recruitment consultation. Sufficient information was provided, families were able to ask questions, understood what the study was about and what would happen if they decided to participate.

YP38: ‘It was really good, it was really useful and it really reassured me and my parents… I was worried that somebody was kind of just going to come in and do a sales pitch, as it were, to us, which probably would have put me off even more’. (Post randomization)

Two parents felt that a lot of resources had been used to explain the study and because they were randomized to the specialist medical care arm, they felt that they had been given information about the Lightning Process that they didn’t need.

P2: ‘I suppose if anything it was slightly overkill, in that we’d already had that information from [the CFS/ME Team] and we’d consented [to contact] to the study, we were up, we were up for it. So it was, it was very nice to see [the research officer] and to have more information and talk about it in more detail, but I think we had already decided that we were going for it’. (Post randomization)

Allocation was discussed in the consultation with the research officer immediately after randomization; seven families felt this was a good thing.

YP38: ‘I think my mum and I were pretty keen to find out once we knew we were going to do it, what group I was in… I was happy to find that out then, I think, because once you’ve said, ‘Yes,’ [to the study] that’s all you’d really worry about’. (Post randomization)

However, one parent would have preferred to receive the allocation by letter.

P33: ‘I think there’s probably a better way: you get sent a letter telling you what it is, so you can deal with your disappointment without somebody sitting there, somebody you don’t know, and you’re not having to hide a reaction you might feel’. (Post randomization)

#### Conflict between interventions

Four parents and children randomized to the specialist medical care and Lightning Process arm described differences between the two interventions in terms of the language and approach used, which meant that the approaches conflicted with each other.

P7: ‘A bit confused. Not quite so bad as we were… I was talking to healthcare professional A, from the ME clinic. I said it feels like we were going to ME clinic and that was fine and we were dealing with it that way, and then we were given this opportunity, which is fantastic, but it felt like after we’d done it, it felt a bit confused as where we were supposed to go from then’. (Post intervention)

P9: ‘It has been a bit confusing, I have to say, because obviously we have got the [Lightning Process practitioners] approach, where, “Right, finally, done this, now you don’t need to do the pacing; you can just go back to school full time.” I think, the physical side of things, YP9 has had to build herself up more rather than just suddenly go back and do that’. (Post intervention)

In light of these findings, we incorporated an explanation of the potential for differences or ‘conflict’ between the two approaches in the patient information leaflet and in the discussion prior to randomization. The research team discussed the issue of conflict with the CFS/ME team and the Lightning Process practitioners, who agreed to respect the different approaches and support parents and children. Parents and children on subsequent courses did not report conflict. Children appeared to cope with differences between the two approaches by using best practice from both to fit with their current health needs.

YP36: ‘Doing them together is okay, but it is quite difficult… But I will definitely keep doing the energy thing until my body is completely healthy again and then once my body is as healthy as any other person my age then I am probably going to try and stick to the Lightning Process after I am totally back at full health’. (Post intervention)

#### Study burden

The number of questionnaires used at follow-up was considered a burden by the majority of children and parents interviewed and observed. Parents felt the timing of questionnaires did not allow time for change, as they were too close together.

P32: ‘The amount of treatment that he’s had, as such, is totally out of proportion, as in small compared to the amount of forms [questionnaires] that have had to be filled in and the amount of information that we’ve had to provide… I just find the whole thing really confusing because they seem, we seem, to kind of get one quite soon after the appointment … I think we’ve had about three; I don’t know how many times they’ve arrived’. (Questionnaire interview)

This resulted in changes to the trial protocol: the six week follow-up questionnaire was dropped and a reduced set of questionnaires (SF-36, Chalder Fatigue and school attendance) was sent with the reminder letter. If participants had still not responded after two weeks, they were telephoned and invited to complete the reduced set of questionnaires over the telephone.

#### Primary outcome

During the study, parents and participants commented that the school attendance primary outcome did not accurately reflect what they were able to do, particularly if they were recruited during, or had transitioned to, A levels during the study. This is because it was not clear what ‘100% of expected attendance’ was. In addition, we were aware of some participants who had chosen not to increase school attendance despite increased activity.

#### Serious adverse events

Two serious adverse events were reported during this part of the study. Both concerned admissions to hospital, one of a child and one of a parent. Neither was related to their involvement in the SMILE study. Both were reported as per routine procedures and no action was necessary by the study team.

## Discussion

It is feasible to recruit children with CFS/ME to a randomized controlled trial comparing specialist medical care with specialist medical care plus the Lightning Process. 44.2% (69/156) of eligible children consented for the research officer to visit them at home: of these, 81.2% (56/69) agreed to participate in the study and were randomized. Children and their parents said that the recruitment and randomization process was acceptable. All families accepted the treatment arm that they were allocated to but 3/28 (10.7%) families in the specialist medical care arm sought the Lightning Process privately; one of these did not receive specialist medical care. In the specialist medical care plus the Lightning Process arm, 3/28 (10.7%) did not attend a Lightning Process course.

### Strengths and limitations

As far as we are aware, this is the first study to attempt to investigate the feasibility and acceptability of a randomized controlled trial investigating the Lightning Process in children. We incorporated qualitative research methods to enable us to investigate issues around randomization and retention, as well as to explore differences between the two treatment approaches. We analyzed audio-recordings of recruitment consultations, which enabled us to improve the balance between interventions in the consultations to improve recruitment and retention. Interviewing parents allowed us to examine difficulties parents might experience with the treatment arms.

Recruitment rates were lower than anticipated, meaning that recruitment continued for longer than planned. This was mainly due to an underestimate of the number of eligible patients at the start of the study (nearly half were not eligible). However, a recruitment rate of 35.9% does not preclude a full study [30].

### Recommendations for a full study

This study suggests that trials involving 17 and 18 year olds need to consider alternative primary outcome measures to school attendance. In addition to the difficulty measuring change in school attendance for those transitioning from GCSEs to A levels, it may be a poor outcome measure for those who do not consider school attendance their primary goal. We suggest that a full study uses other primary outcomes, such as the SF-36 or the Chalder Fatigue Scale and uses school attendance as a secondary outcome. Future studies should consider using different patient information sheets for children aged 12 to 14 years than those used for older teenagers. The word ‘interview’ should be defined more fully in future information sheets or possibly a different word or description used, perhaps ‘a discussion with a researcher to get your views’. We have demonstrated that it is possible to improve outcome data collection using a variety of strategies, including telephone follow-up, and these would need to be implemented in a full study. In addition, we would recommend protocols to identify increased disease activity during the course of the study, such as those used in the PACE trial [31].

### Ethical acceptability

We made an assessment of whether a full study could operate ethically. We suggest that, provided appropriate measures remain in place or are improved (for example, the patient information sheet) there is no reason to suppose that a full study could not be ethically sound. This is not to anticipate the outcome of reviews of relevant research ethics committees that would be necessary before starting a full study.

The key areas for ethical consideration in any research study with human participants can be categorized as (i) respecting individuals’ autonomy; (ii) welfare of participants; (iii) wider societal interests [32].

i. Respect for individuals’ autonomy (and by implication respecting potential participants’ choices) [33] was inherent in the study design by use of: clear information; recruitment and consent procedures that ensured voluntariness; and confidentiality agreements. The procedures for obtaining consent varied according to age group, to account for the corresponding differences in capacity [34]. To participate, parents and patients over 16 years old had to give consent for their own involvement. Those under 16 years provided assent and their parent consented on their behalf. In all cases, the research process involved a patient-parental partnership so parents were fully aware of their child’s participation. We recommend that a full study should operate according to a similar policy, or a policy of getting both assent and parental consent for all patients. Comprehensible and accessible information is essential for ensuring that both consent and assent are valid, since the participant is making a choice based on that information. Feedback suggests the information sheets could be improved to be more age-appropriate with a better description of interviews.

ii. Participant welfare was safeguarded throughout the study, and the level of inconvenience minimized. For example, the number and length of questionnaires were reduced, as we would recommend for a full study. No patient was deprived of any treatment they would normally have been offered, and we suggest that this should be the same for a full study.

iii. Any research study involving participants must be ethically justifiable in terms of its potential wider impact and value. We consider there to be a strong moral case for conducting a full-scale study into the effectiveness of the Lightning Process as a treatment for CFS. This is based on the needs of children and teenagers with CFS, the lack of evidence for the best methods of treatment, and the fact that participating in the trial presents relatively little burden for patients and parents.

## Conclusion

Conducting an RCT to investigate an alternative intervention in children with CFS/ME is feasible and acceptable, even if the intervention is controversial and highly publicized. This study demonstrates the importance of feasibility studies before a full study [35]. The qualitative methodology enabled us to understand problems with study overload and reduce the number of questionnaires used; and to understand conflict between the two interventions, which required additional information in the patient information sheet. Neither of these problems would have been easily detected otherwise. This feasibility study led to several changes to the proposed methodology of the full study. Future research is needed to provide more information on the Lightning Process and differences between the Lightning Process and specialist medical care.

### Interview topic guide

#### These questions are to be used as prompts to ensure that all important areas are covered

Welcome, introduction, stress confidentiality. Discuss consent, sign form or check continues to be happy with consent.

After assessment and before randomization

1) **Can you talk me through your initial appointment with the research nurse?**

Prompts: What was said, did you understand what was being said? Feelings?

2) **What were your initial thoughts about the study?**

Prompts: What did you think when you were told about it? Feelings? Worries? Expectations?

3) **Did you know anything about the Lightning Process before this initial appointment (for first interview only)?**

Prompts: How? Who? What did you think? What information?

4) **What did you think about the information you were given about the study?**

Prompts: What information did you get – oral and written? Did you read it? Understand it? Did it give you enough information? Too much? Were there things you thought they had forgotten to include?

5) **Have you found out any information about the Lightning Process since?**

Prompts: Why? How? What did you find? What did you think?

6) **What are your thoughts at this stage on taking part or not? Why?**

Note: Stress that they are not being asked at this stage but that we want to gauge their thoughts, stress also that it makes no difference to the interviewer.

7) **If you were to take part, would you have a preference for one of the interventions?**

Prompts: Why? Issues over participation? Engagement? What would you do if allocated the other intervention?

8) **What do you think about having treatments allocated at random, that is, by chance?**

Prompts: Why is it done? How do you feel about this way of deciding what treatment you’ll get? Is there a better way? Do you think you’ll be happy to be randomized? Do you think you’re likely to get one intervention rather than the other? Why?

9) **You have now done some questionnaires at follow-up. What did you/your child think about the questions you were asked?**

Prompts: Were there any particularly difficult questionnaires? What did you think about the HADS/ POMS inventory? Would you leave some questionnaires out? Other areas that should be covered?

After randomization and before interventions

1) **Can you tell me what happened when the research nurse visited and explained about randomization?**

Prompts: What did she say? Understandable? What did you think? Did you understand what was going to happen?

2) **What did you think before randomization?**

Prompts: Were you happy with the process? Did you understand what was going to happen and why?

3) **Did you agree to randomization or not? Why?**

4) **What did you think when you got your intervention allocation?**

Prompts: How did you feel? Was it what you expected? Wanted? Expectations of intervention? What have you done since then?

5) **You have now completed some questionnaires at follow-up. What did you/your child think about the questions you were asked?**

Prompts: Were there any particularly difficult questionnaires? What did you think about HADS/POMS inventory? Would you leave some questionnaires out? Other areas that should be covered?

After intervention

1) **Tell me about the intervention you received?**

Prompts: What happened? What was good/bad? What would you change? Venue? Structure of sessions? Language used? Was it as expected?

2) **Do you think you/your child have/has learnt anything from it, if so what?**

Prompts: About CFS/ME, themselves, self-management?

3) **What has happened after the intervention?**

Prompts: How have you/they done? What are you/they doing? Feeling?

4) **What do you think now about being randomized?**

Prompts: Would you do it again? What do you think about the study for others?

5) **You have now completed some questionnaires at follow-up. What did you/your child think about the questions you were asked?**

Prompts: Were there any particularly difficult questionnaires? What did you think about the HADS/ POMS inventory? Would you leave some questionnaires out? Other areas that should be covered?

## Abbreviations

CFS: Chronic fatigue syndrome; EQ-5D: EuroQol 5D; GET: Graded exercise therapy; HADS: Hospital anxiety and depression scale; LP: Lightning Process; ME: Myalgic encephalomyelitis; NICE: National Institute of Health and Care Excellence; P: Parent; POMS: Profile of mood states; RCT: Randomized controlled trial; SMILE: Specialist medical intervention lightning evaluation; WPAI:GH: Work productivity and activity impairment and general health; YP: Young person.

## Competing interests

Dr Crawley is a medical advisor for the Association for Young People with ME (AYME), and for the Sussex & Kent ME/CFS Society.

## Authors’ contributions

EC conceived the study, participated in the trial design and coordination and drafted the manuscript. NM designed the qualitative methodology and supervised the qualitative data analyses. LB conducted the qualitative interviews and analyzed the qualitative data. DJ and KW recruited the participants and contributed to study management and interpretation of results. SMC conducted the statistical analyses. ZD provided ethical advice and reviewed the protocol at each stage. AM contributed to the study design and interpretation of the results. All authors contributed to and approved the final manuscript.
